# Towards Customized Footwear with Improved Comfort

**DOI:** 10.3390/ma14071738

**Published:** 2021-04-01

**Authors:** Rafaela Teixeira, Carlos Coelho, João Oliveira, Joana Gomes, Vera Vaz Pinto, Maria José Ferreira, João Miguel Nóbrega, Alexandre Ferreira da Silva, Olga Sousa Carneiro

**Affiliations:** 1 IPC—Institute for Polymers and Composites, University of Minho, 4800-058 Guimarães, Portugal; b8029@dep.uminho.pt (R.T.); joao.oliveira@dtx-colab.pt (J.O.); mnobrega@dep.uminho.pt (J.M.N.); 2 Atlanta—Componentes Para Calçado, Lda, Marco de Simães, 4615-414 Macieira da Lixa, Portugal; producao@atlantasteps.com; 3 CTCP—Portuguese Footwear Technological Centre, 3700-121 São João da Madeira, Portugal; joana.rita@ctcp.pt (J.G.); vera.pinto@ctcp.pt (V.V.P.); mjose.ferreira@ctcp.pt (M.J.F.); 4 CMEMS-UMinho—Center for MicroElectroMechanical Systems, University of Minho, 4800-058 Guimarães, Portugal; asilva@dei.uminho.pt

**Keywords:** shoe comfort, biomechanical customization, Fused Filament Fabrication, 3D printing, graded properties, cellular structure

## Abstract

A methodology enabling the customization of shoes for comfort improvement is proposed and assessed. For this aim, 3D printed graded density inserts were placed in one of the critical plantar pressure zones of conventional insoles, the heel. A semi-automated routine was developed to design the 3D inserts ready for printing, which comprises three main stages: (i) the definition of the number of areas with different mesh density, (ii) the generation of 2D components with continuous graded mesh density, and (iii) the generation of a 3D component having the same 2D base mesh. The adequacy of the mesh densities used in the inserts was previously assessed through compression tests, using uniform mesh density samples. Slippers with different pairs of inserts embedded in their insoles were mechanically characterized, and their comfort was qualitatively assessed by a panel of users. All users found a particular pair, or a set, of prototype slippers more comfortable than the original ones, taken as reference, but their preferences were not consensual. This emphasizes the need for shoe customization, and the usefulness of the proposed methodology to achieve such a goal.

## 1. Introduction

From a user perspective, comfort is one of the most relevant features in footwear assessment [1]. The demand for improved comfort is increasing as users become aware about its beneficial effects on health and wellbeing [2]. However, comfort is subjective and, therefore, a difficult attribute to define. The perception of comfort is highly user dependent [3]. Nevertheless, there are some parameters that can be related to the perceived shoe comfort, such as interior softness, thermal comfort (temperature and humidity), flexibility, weight, damping capability, heel zone impact absorption, and plantar pressure distribution, among others [1].

Several studies correlate the increase of plantar pressure with pain and discomfort [4], but up to now there is no clear idea of what pressure distribution most favors shoe comfort perception or how to deal with the non-uniform plantar pressure distribution [5]. Moreover, plantar pressure depends on the weight acting and on the contact area, so any change in weight or reduction of the contact area will affect it [6,7]. The importance of this parameter led to the development of devoted measuring/monitoring systems, which are of two main types: (i) platform systems that enable the acquisition of data of barefoot people, removing, therefore, any influence of the shoes in the exerted plantar pressure; (ii) in-shoe systems, which are composed by flexible sensors inserted in the shoe interior thus enabling data acquisition during gait [8,9,10].

Researchers have been searching for new materials and new ways to build shoes in order to balance the plantar pressure distribution [4]. However, since comfort is based on personal perception, a universal solution seems unfeasible. It is under this premise that additive manufacturing and rapid prototyping processes become valuable, enabling a certain degree of customization. Currently, Fused Filament Fabrication (FFF) seems to be the most adequate technique to solve this problem, mainly due to the geometrical freedom it provides, when compared to the conventional manufacturing processes, and to the vast range of materials that it can use. Owing to its working principle, it enables the production of components presenting a set of characteristics and functionalities that were not achievable before. In addition to the geometrical freedom, it also enables the combination of several different materials in the same component, when multi-material FFF techniques are adopted [11,12]. In this case, flexible and rigid materials, opaque and transparent materials, or different colors of the same material, are just a few examples of the combinations that can co-exist in a single printed part.

Despite all these possibilities, the need for innovative industrial solutions and the advances in material science led to the development of the so-called functional graded materials (FGM) [13]. In the last years, FGM turn into a new category of engineering materials, being the focus of several researchers. FGMs present gradual varying properties, which are a consequence of varying structure, microstructure, chemical composition, or phase distribution [14]. Mechanical properties, such as the Young’s modulus, Poisson’s coefficient, density or thermal expansion coefficient may vary in a gradual and continuous manner in these type of materials [15]. This feature enables the matching of materials properties to a local part request [16,17], or to minimize the amount of material required to get a specific performance [18]. FGM may be designed according to one of the two following principles: continuous structures, having a continuous gradient between the two materials/properties; or non-continuous structures, where there is a discrete variation of the property/material along several homogeneous interfacing layers [15,19]. These materials can also be classified according to the way grading is promoted: variation of chemical composition, or variation of the spatial structure, e.g., variation of the porosity or of the structure lattice [20].

In the context of shoe comfort, Tang and co-workers [21] recently proposed a new design method, specially devoted to diabetics, for optimizing the stress distribution in the foot-insole contact surface. For this purpose, they used functional gradient structural properties in order to increase the foot-insole contact area, thus decreasing the peak contact area. This approach consisted in the development of modular elements, having different elastic moduli, which were combined through an optimization scheme to solve the problem of a specific user (initial contact area/pressure distribution).

The objective of the present research is slightly different since it focuses on the development of a methodology that enables to produce, through a simple and automatic way, insole inserts having different densities distributions that can be used to increase comfort. Two main differences can be depicted: (i) the graded density zones are inserts of the insole, enabling, therefore, a simple and fast way to customize it; (ii) the graded density inserts have a continuous structure, automatically generated, not being composed by a juxtaposition of discrete modular elements.

## 2. Methodology

**Figure 1:**
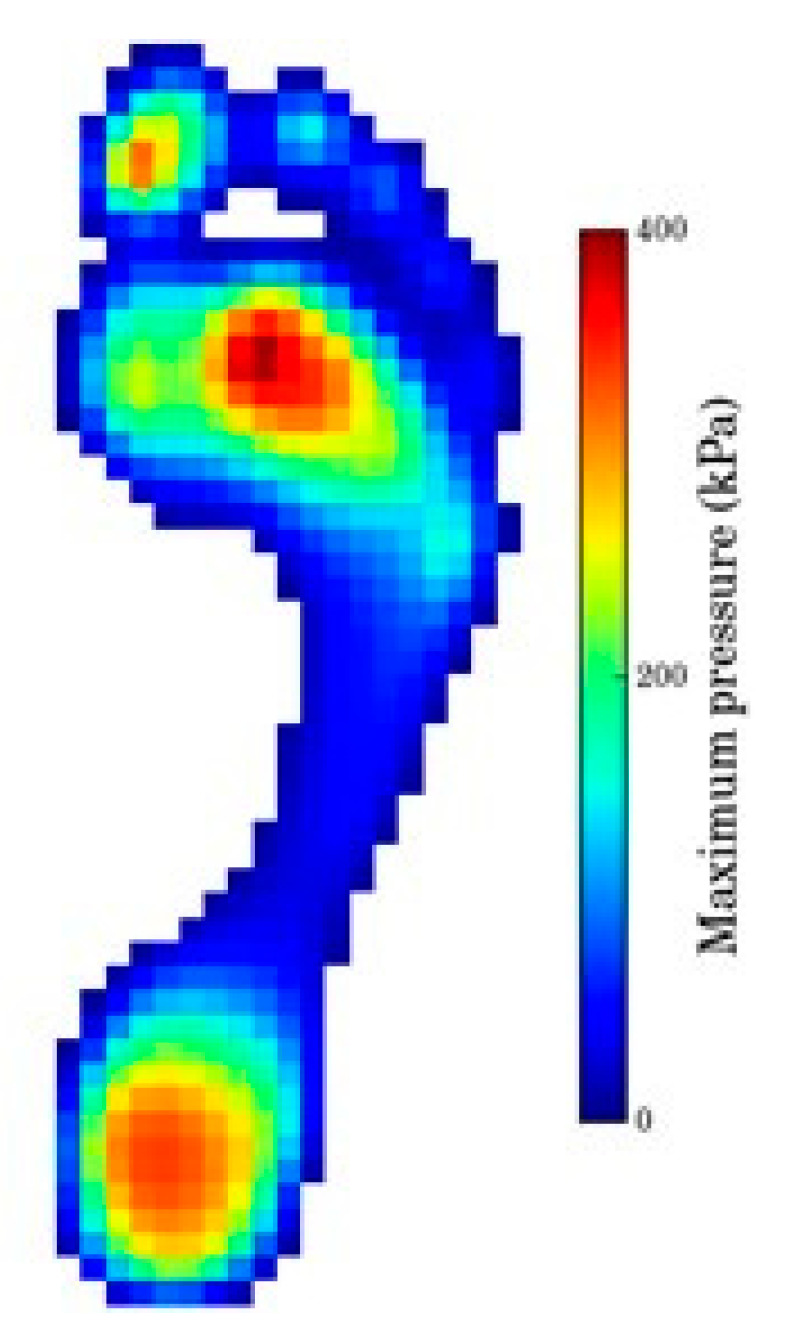
Typical plantar pressure distribution (licensed under the Creative Commons Attribution-Share Alike 3.0).

Figure 1 shows a typical plantar pressure distribution in static condition (standing), where three high pressure zones are easily discernible, namely the heel, forefoot and hallux.

Independently of the specific solution to be adopted for promoting a higher comfort, it is clear that it has to act on the three critical zones identified. The hereby proposed strategy is to create insole inserts for these zones. This design decision brings two advantages: (i) it may be used in commercially available shoes, adapted to each individual, easily providing customized solutions; and (ii) for the purpose of this research, it enables testing different solutions in the same pair of shoes. In fact, the customization of the insole focuses on the FFF produced inserts that are placed in the critical zones of conventional insoles. This way, the negative effect of the low printing speeds, typical of FFF techniques, will be minimized since the majority of the insole will still be produced by injection molding.

**Figure 2:**
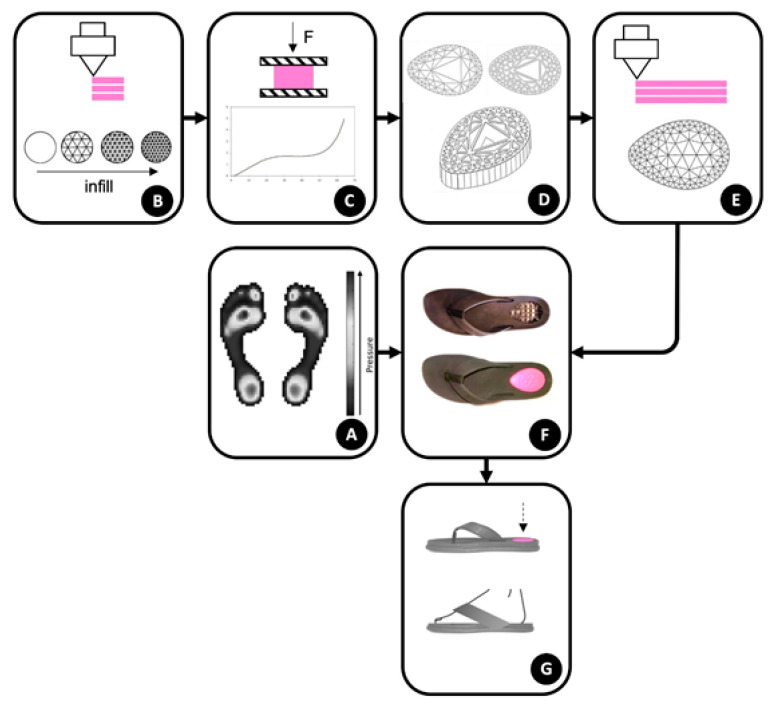
Methodology followed in the present work: (**A**)—Identification of critical plantar pressure zones; (**B**)—Printing of uniform mesh density structures; (**C**)—Compression tests of the previous structures; (**D**)—Generation of graded density structure inserts; (**E**)—Printing of the inserts; (**F**)—Production of prototype slippers (laser cut of insole, slipper assembly and insert placement); (**G**)—Comfort assessment of prototype slippers (dynamic impact tests and assessment by a panel of users).

The geometric freedom provided by FFF technique is used to produce inserts with graded flexibility, as a result of an internal filling built with a graded mesh density. Keeping in mind that the objective is to understand which type of structure provides the best comfort, we decided to carry out this research solely focused on the heel zone, since the final conclusions will provide guidelines applicable to the remaining critical plantar zones. The methodology proposed is illustrated in Figure 2. Each one of the main stages, namely the characterization of uniform mesh density structures, generation of graded density structure inserts, and production and assessment of prototype slippers, will be addressed in detail in the next sections.

## 3. Study on Uniform Mesh Density Structures

### 3.1. Material, Geometry and Mesh Densities

The design of functional graded structures of the heel inserts was preceded by a simpler research on the effect of the mesh density on the compression behavior of uniform mesh density samples. The expected differences in the compression behavior of the printed samples/structures obtained in this way will enable us to assess the potential of using a single material to obtain different structure properties. For this sake, the capabilities of the Simplify3D slicer software (available at https://www.simplify3d.com/, accessed on 29 March 2021) were used, by selecting the triangular mesh and different filling densities for the internal structure of the samples. The triangular geometry was selected since it facilitates the automatic generation of graded density meshes, as will be described in Section 4.

**Figure 3:**
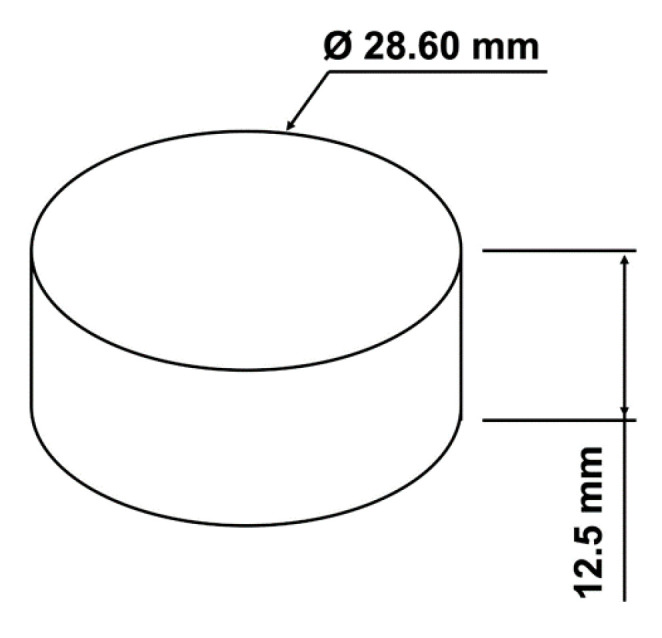
External geometry and dimensions of the compression test samples used, according to ASTM D575 [22].

Standard ASTM D575 [22] was used for the compression tests carried out with cylindrical samples, with the dimensions illustrated in Figure 3.

**Figure 4:**
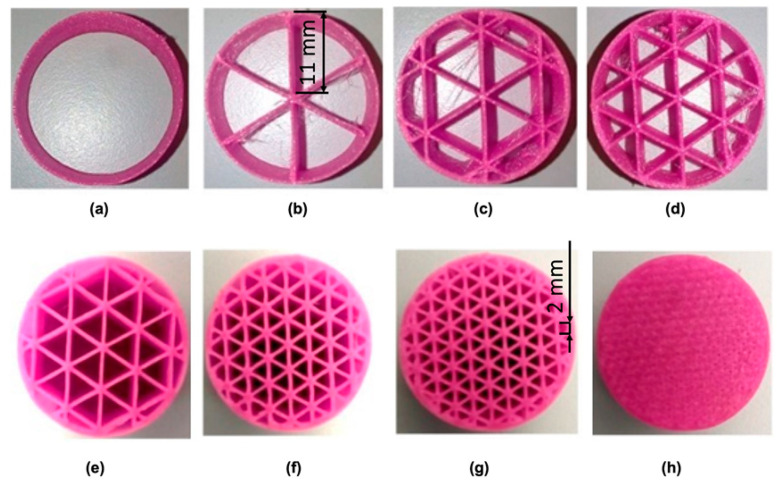
Printed samples, for compression tests, with different filling densities (triangular mesh): (**a**) 0%; (**b**) 10%; (**c**) 15%; (**d**) 20%; (**e**) 25%; (**f**) 40%; (**g**) 50%; (**h**) 100% (dimensions in mm).

The samples were printed in an FFF 3D printer, original Prusa i3 MK3 (Prusa Research, Prague, Czech Republic), using Filaflex, a polyurethane based thermoplastic elastomer filament, with Shore A 82. This filament material was selected due to its similarity to the material used to produce the slippers insole that was used as a base structure in the present case study (see Section 5). The test samples were produced without top and bottom layers, and with a single thin (0.24 mm thickness) side wall, in order to minimize the influence of the geometry contour on the behavior of the internal structure. Several filling densities were tested (0, 10, 15, 20, 25, 40, 50 and 100 %), as illustrated in Figure 4, providing meshes with different densities. When the filling density varies from 10 % to 50%, the resulting cell size varies from around 11 mm to 2 mm (see Figure 4). The height of the samples (and cells) after printing is constant and around 12.5 mm (see Figure 3).

### 3.2. Compression Tests

Compression tests were carried out in a universal mechanical testing machine, Instron 5959 (Instron, Norwood, MA, USA). A maximum compression head displacement of 8 mm (strain of 64%) and a test speed of 50 mm/min were selected.

**Figure 5:**
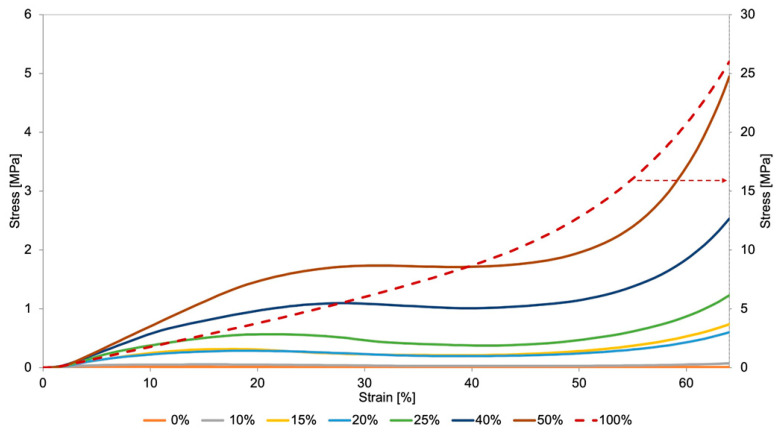
Stress—strain curves of the different types of samples tested in compression. (Note: the stress axe on the right should only be used for the 100% case).

The stress (*σ*)—strain (*ε*) curves obtained in the tests are illustrated in Figure 5, being the elastic modulus (*E*), maximum stress (*σ_max_*), i.e., stress at yielding, and strain and displacement at the maximum stress, listed in Table 1.

As expected, increasing filling densities results in higher structure stiffness (higher modulus and maximum stress), i.e., with the same material, different meta-properties for the structure are obtained, which may vary within a wide range. This preliminary study provides, therefore, a relationship between cell size and structure stiffness, an important information to tune local properties of structures having graded mesh densities.

**Figure 6:**
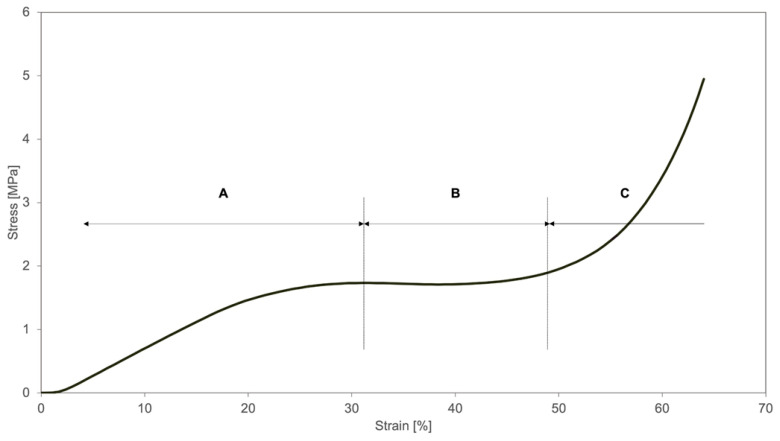
Typical zones of the behavior of the structures during the compression tests (in this case, the 50 % filling density is shown): A—elastic deformation of the material; B—small deformation of the structure with progressive collapse due to vertical walls buckling; C—full collapse of the structure with inherent densification (compression of a compact structure).

The typical behavior observed during the compression tests is illustrated in Figure 6, where three different regions are identified:

- the linear region of the stress-strain curve, where the stress increases proportionally to the strain imposed to the material;
- region where the structure deformation takes place, due to the buckling of the vertical walls, corresponding to a plateau/quasi-plateau region where the strain increases at almost constant stress (the stress at yielding);
- densification region where the stress starts again to increase, due to the full collapse or crushing of the structure. In these conditions, an almost compact structure is, therefore, being compressed.

In the present study the cellular structures obtained are of 2D type, but their behavior is similar to that of 3D cellular materials/structures such as, for example, cork [23], or Polyurethane foams [24,25,26].

In terms of the envisaged application, the inserts will have internal structures capable of withstanding the local plantar pressures without collapsing. In fact, the collapse of the structure would result in very high stiffness and almost no impact absorption capability, inducing discomfort. Therefore, these stresses should not exceed the maximum ones previously identified for each mesh density.

The maximum plantar pressure depends on several variables, as person’s weight and foot/floor contact surface, gait attitude, footwear and type of floor and inclination [27]. However, this value is known to be lower than 1 MPa for both static (standing) and dynamic (walking and running) conditions [27]. The results obtained in the compression tests of the uniform mesh density structures (Table 1), revealed maximum admissible stresses in the 0.05 to 1.7 MPa range (values corresponding to 10% to 50% filling densities, respectively), i.e., a range adequate for insole applications. Therefore, it may be concluded that the base material (Filaflex) and the range of mesh densities tested were successfully selected.

## 4. Structures with Mesh Density Gradient

After determining the effect of using different filling densities, the next step consisted in the generation of inserts with graded mesh density structures adjusted to the plantar pressure distribution. A 12 mm height, similar to that of the compression test samples, was selected for the inserts. The mesh density, variable in this case, resulted from cells sizes between 2 and 11 mm, i.e., in the range previously tested.

**Figure 7:**
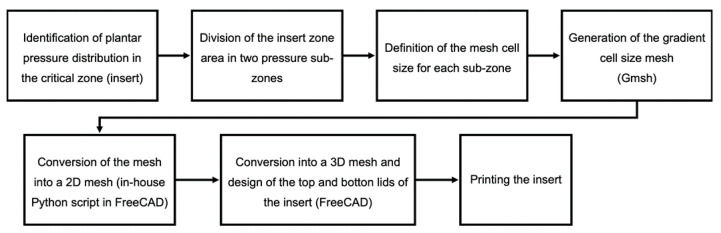
Sequence adopted in the design of the gradient property insole inserts.

**Figure 8:**
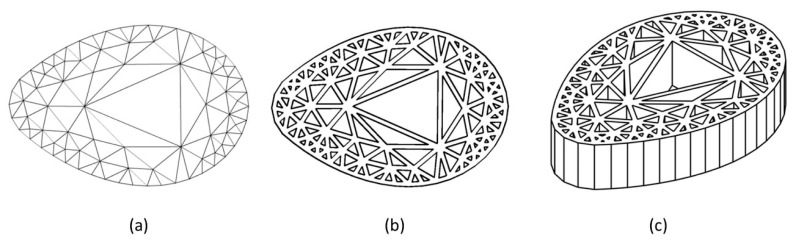
Main steps of the insole insert generation process: (**a**) gradient predefined mesh; (**b**) 2D mesh with predefined wall thickness; (**c**) 3D mesh.

The first attempts to design inserts with two different mesh densities zones, directly from Simplify3D software, was unsuccessful. More specifically, despite being possible to define sub-zones with different filling densities, the software created a boundary wall at their interface. As a consequence, the mesh was not continuous and the boundary wall would affect the compression behavior of the structure, mostly for the larger cell size cases (lower mesh densities). Furthermore, the cell size varied in a discrete way, with an abrupt change at the sub-zones’ boundary. According to the smooth plantar pressure distribution (see Figure 1), variable cell sizes, without sharp transitions, seems to be more adequate. Nevertheless, manually drawing a part with graded mesh density would be labor- and time-consuming. To overcome this drawback, a computer routine was developed to automatically generate the inserts with graded mesh densities. Having in mind the small dimensions of the insole critical zones, only two regions were considered for the definition of different mesh densities (cell sizes). The sequence of operations required to get a gradient density insert is illustrated in Figure 7. The process starts by the analysis of the critical zone plantar pressure distribution. Based on this and on the geometry and dimension of the insert, the location and geometry of the boundary between the two different cell size sub-zones is defined. Then, and considering the local absolute values of pressure, the cell size of each sub-zone is outlined. Subsequently, the insert area is triangularized with the predefined mesh densities defined (Figure 8a), using the mesh generator Gmsh [28]. This mesh is then imported to FreeCAD [29], in which a Python script was programmed to generate the 2D geometry, with a predefined mesh wall thickness (Figure 8b). This 2D geometry is subsequently extruded to create the internal structure of the insert (Figure 8c). The insert geometry creation is finalized by adding the top and bottom layers in FreeCAD. After these steps, the inserts will be ready for printing.

**Figure 9:**
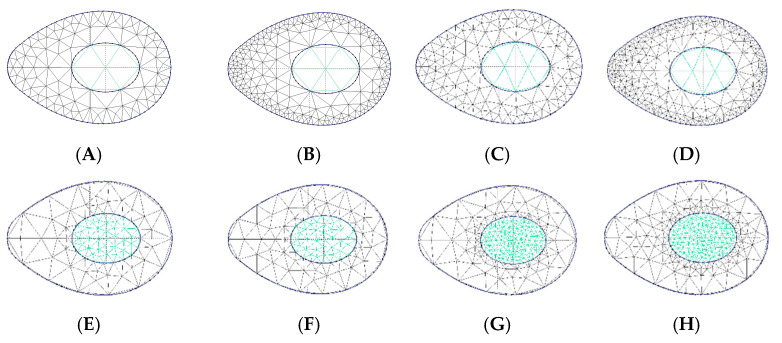
Heel zone inserts printed with different graded mesh densities: inserts (**A**)–(**D**) with bigger cell size (lower mesh density) in the middle and smaller cell size (higher mesh density) in the periphery; inserts (**E**–**H**) with the reverse mesh density distribution.

**Figure 10:**
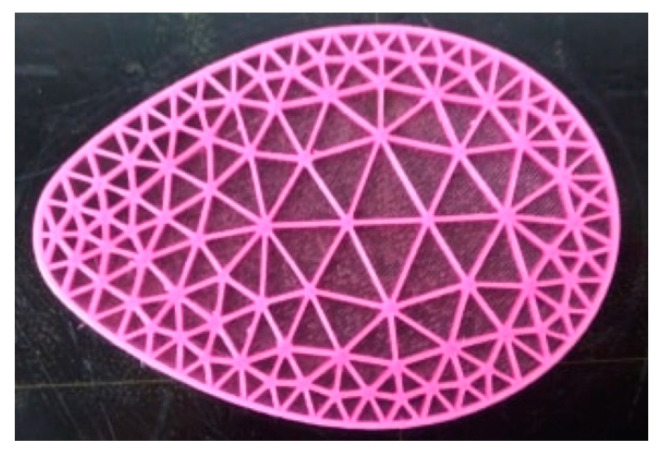
Example of a heel zone insert printed without top lid (solely intended to show its internal structure).

Using the above methodology, the eight different types of heel zone inserts illustrated in Figure 9 were designed and printed. In this set of inserts two main types were considered: those with bigger cell size (lower mesh density) in the middle and smaller cell size (higher mesh density) in the periphery (Figure 9, inserts A–D), and the reverse (Figure 9, inserts E–H). Additionally, for each of the above referred sets several cell sizes were used in each sub-zone. In the examples shown, the line that separates the two sub-zones was used to define the location of the boundary but does not exists physically in the printed models, as can be seen in the example shown in Figure 10.

**Figure 11:**
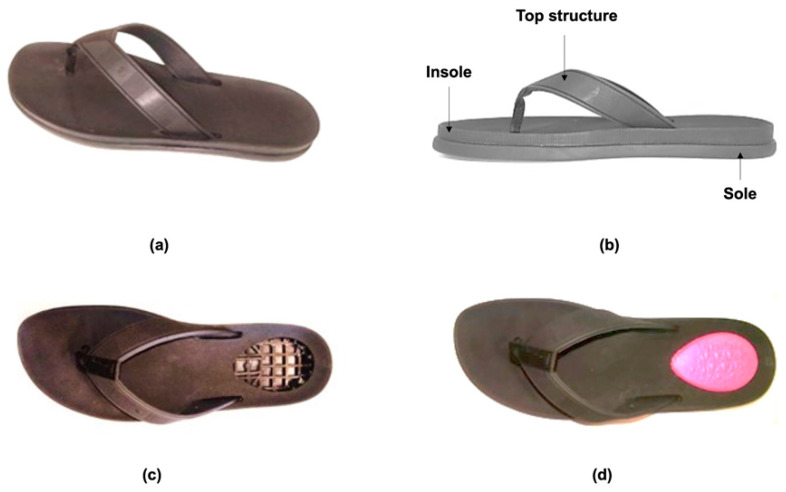
Slippers used in the case study: (**a**) original TPU slipper; (**b**) structure of the slippers; (**c**) prototype slipper with a cut in the insole heel zone for insert placement; (**d**) prototype slipper with insert.

Pairs of each insert shown in Figure 9 were printed with the same filament and printer that were used in the production of the uniform mesh density samples. One of these printed inserts can be seen in Figure 11d.

## 5. Case Study

### 5.1. Original Shoes and Prototypes

The original shoes used for comfort assessment are the slippers shown in Figure 11a,b. These encompass three components, namely the sole, the insole, and the top structure for foot prison, positioning, and holding. The sole and insole are assembled using an adhesive, being the upper structure fixed between these two components through tabs trapped in cavities defined at their interface. The insole was produced by injection molding in a TPU that has a similar hardness to that of the Filaflex filament. The prototype shoes, illustrated in Figure 11c,d, were produced by laser cutting the insoles, at the heel zone, before assembling the three components.

### 5.2. Comfort Assessment

Prototype slippers, with pairs of inserts placed in their insoles, were subjected to a dynamic impact load test frequently used to assess the cushioning properties of sport shoes insoles [30]. For this sake, a Zipor equipment, model EL-44, was used. An 8.5 kg impactor (striker) was dropped vertically, from a 50 mm height, onto the test samples. The deceleration of the impactor was measured with an accelerometer attached to the impactor during impact. The height of the first rebound of the impactor was also measured and the energy return calculated. The results obtained are shown in Table 2, where the reference case corresponds to the original TPU slippers.

The reference TPU slippers present the higher value of maximum deceleration. Thus, they are expected to promote the lowest comfort when subjected to an impact. This was anticipated since the insole is totally compact. For the prototype slippers, this value depends on the average density of the inserts and its spatial distribution. Another property shown in Table 2 is the energy return, which is a measure of the structure resilience and its capability to absorb the impact energy. The lower values of this property are those corresponding to inserts A to D, i.e., those having a lower mesh density in the middle (see Figure 9). According to the shoe experts involved in this study, for casual shoes the recommended values for maximum deceleration and energy return should be in the 120–160 ms^−2^ and 32–50% ranges, respectively. Having this into consideration, it may be concluded that all the prototype slippers perform better than the reference ones in what concerns to the maximum deceleration, and that the majority also perform better when the energy return is taken into account. In this last case, only inserts G and H have a lower performance than the reference slippers. Therefore, at this stage the inserts that show the best cushioning properties are E and F, presenting the lower values for maximum deceleration and a good resilience (adequate values of the energy return).

In order to verify if there is a relationship between these mechanical properties and comfort perception, the prototype slippers and the reference ones were also tested by a panel of users. The main objective was to determine which of the two main types of structures provides higher comfort and how they compare with the original slippers (lower, similar, or higher comfort). The main results of this study are shown in Table 3.

The assessment results were not consensual, and two main groups of users can be identified: those that select as more comfortable the prototype slippers with inserts A to D, valuing their softness and impact absorption capability; and those that valued stability, selecting the prototypes with inserts E to H. As referred in the Introduction, comfort is a subjective concept, which perception is highly dependent on the user. This was confirmed in the present study that clearly strengthens the need for shoe customization. Anyway, all the users considered their specific preferences as more comfortable than the reference (original) slippers, putting in evidence the potential of the strategy developed in this work to tailor shoes insoles for comfort improvement.

## 6. Conclusions

In this work, a methodology enabling the customization of shoes for comfort improvement was proposed and assessed. The first step consisted in a study where the selected material, a TPU filament, was employed to print several different uniform mesh density structures, obtained through the use of different printing filling densities. This study showed that the compression behavior of the different structures varied significantly with the filling density, presenting maximum (yield) stresses between 0.05 to 1.7 MPa, and that the smaller cells structure was capable to withstand the maximum typical plantar pressures without collapsing. Thus, it was concluded that the material and the cell size range tested were adequate for shoe insole applications.

The next step consisted in generating and printing insole inserts comprising graded mesh density structures for the heel zone, one the of the critical zones in terms of plantar pressure. Graded properties were considered relevant due to the typical patterns of plantar pressure distribution. The graded density structures were generated with an in-house developed methodology that enabled a continuous variation of the cell size and, hence, graded mechanical properties. Eight different pairs of inserts were printed by the FFF technique and embedded in TPU slippers insole. For this sake, the original insoles of these shoes were laser cut, at the heel zone, to create a cavity for the 3D printed inserts. These inserts were of two main types: those having a lower mesh density in the center and higher in the periphery, and those having the opposite mesh density distribution.

The prototype slippers (those having inserts embedded in their insoles) and an original pair of slippers (the reference case), were mechanically characterized through dynamic impact load tests, and their comfort was assessed by a panel of users. The properties determined in the impact tests were the maximum deceleration, related with the capability to promote comfort, and the energy return, a measure of the resilience, i.e., the impact absorption capability. It was concluded that all the prototype slippers tested had a better performance than the original ones in what concerns to the maximum deceleration. In fact, this value was always lower than the reference value (around 270 ms^−2^) and reached a minimum of around 160 ms^−2^ in inserts having a higher density in the center (recommended range for casual shoes: 120-160 ms^−2^). Concerning the energy return, the recommended range is 32–50 %. In the majority of the cases, both the prototype slippers and the reference ones presented values in this range. The only exceptions were two prototype shoes, in which the inserts had very high mesh density in the center and low mesh density in the periphery.

The prototypes slippers were also tested by a panel of users, for comfort assessment purposes. All the users found at least a pair of prototype slippers more comfortable than the original (reference) one. However, their preferences varied. Furthermore, the most promising inserts in terms of mechanical performance were not, definitively, the predominant choice of the users. This emphasizes the subjectivity of comfort perception and the need for shoes biomechanical customization, which can be done via the proposed methodology.

In conclusion, slipper or any other type of shoes, can be produced with a special insole (cut in the critical plantar pressure zones) where inserts selected from a set of pre-produced ones can be used. In fact, the present study showed that there is no ‘best insole’ since the preferences of the users varied.

## Figures and Tables

**Table 1:** Results of the compression tests.

	Filling Density (%)							
	0	10	15	20	25	40	50	100
Elastic modulus, E (kPa)	2.2	10.7	31.5	35.2	53.0	65.8	81.2	199.5
Maximum stress, *σ_max_* (kPa)	17.4	52.3	313.8	286.9	567.1	1094.6	1734.3	25,997.1
Strain at *σ_max_* (%)	3.0	15.0	17.7	18.4	21.0	27.7	31.7	64.0
Displacement at *σ_max_* (mm)	0.4	1.9	2.2	2.3	2.7	3.5	4.0	8.0

**Table 2:** Results of the dynamic impact tests obtained with the reference slippers and the eight pairs of prototype slippers.

		Maximum Deceleration (ms^−2^)			Energy Return (%)		
		Left	Right	Average	Left	Right	Average
Reference		270.61	277.11	273.86	50.06	48.84	49.45
Inserts	A	224.67	226.45	225.56	39.05	37.15	38.10
	B	215.02	218.22	216.62	40.98	37.83	39.41
	C	220.95	204.88	212.92	38.74	40.98	39.86
	D	210.15	215.58	212.87	43.72	43.85	43.79
	E	161.80	164.93	163.37	45.44	45.52	45.48
	F	168.19	162.51	165.35	44.37	43.32	43.85
	G	237.62	228.82	233.22	55.66	55.38	55.52
	H	250.33	238.17	244.25	56.31	54.67	55.49

**Table 3:** Information collected in the tests performed by a panel of users.

User	Gender	Weight Range (kg)	More Comfortable Inserts (Justification)	Less Comfortable Inserts (Justification)
1	M	61–70	E and F (higher stability)	
2	M	61–70	C (softer)	
3	M	81–90	A (softer) and H (stable)	
4	M	81–90	B (better heel impact absorption)	
5	M	71–80	A to D (better heel impact absorption) and E	F to H (too rigid)
6	M	81–90	C and D (lower heel pressure)	G and H (higher heel pressure)
7	M	61–70	A to D (better impact absorption)	
8	M	81–90	G and H (more rigid)	
9	F	61–70	A (absorbs better the impact)	H (too rigid. Similar to the reference slippers)
10	M	61–70	C and D (softer)	G and H (too rigid)
11	M	71–80	F (rigid; similar to the reference slippers)	A and B (not stable)
12	M	61–70	A to D (softer)	
13	M	71–80	A to D (softer)	
14	F	61–70	A to D (softer)	

## Data Availability

Data is contained within the article.
